# New challenges in the new year for Phcog Mag.: 5 years of quality publication

**DOI:** 10.4103/0973-1296.75865

**Published:** 2011

**Authors:** K. K. Mueen Ahmed

**Affiliations:** *Managing Editor, Pharmacognosy Magazine, Bangalore, India*

When we started in the year 2005, it was very anxious how things might turn out with the new journal started during that time. I am very happy to present you a report on Phcog Mag. for the last 5 years. The journal seems to be flourishing and expanding day by day. High-quality papers are being published in every issue and the issue is being launched on time. It was a remarkable amount of growth when we merged with Medknow Publications and Media Pvt. Ltd. in the year 2009. We are now having an online manuscript submission system (Journalonweb) through which peer review process is much faster. Our site is fully dynamic and the papers being published are now available in multiple formats such as PDF, Full text, ePUB, Sword repository plugin, eBook, etc. We have received only positive comments about the new cover that was introduced last year. So, this will remain in place for the foreseeable future. We have summarized the achievement as highlights of the year 2010[Table 1]. We are very thankful to Medknow Publications and Media Pvt. Ltd. for their excellent work in making our journal reach good heights.

**Table:** Highlights of the year 2010

PubMed indexing Online manuscript submission system (Journalonweb) Articles published in multiple format Issues are now available in eBook format References are downloaded in different citation format New page layout and journal cover New Editorial Board Inclusion of more than 200 new reviewers, etc. Speedier publication process Swift decisions and faster peer review

## QUALITY ARTICLES

**Figure 1:**
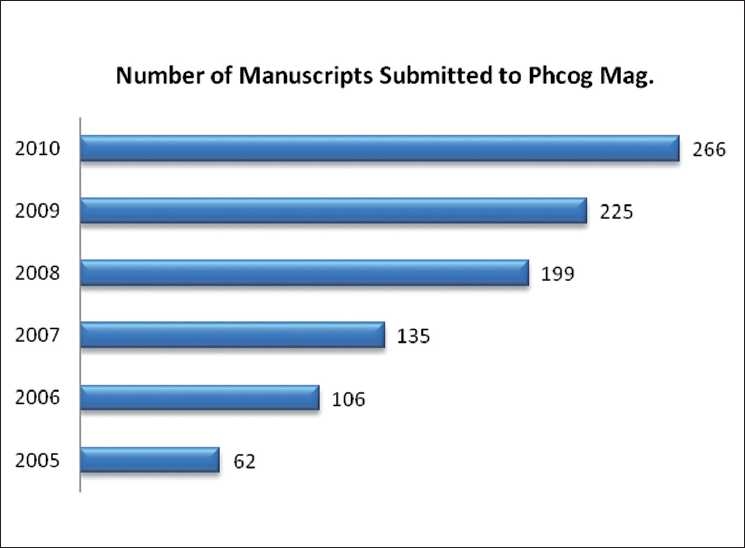
Number of submissions in the past 5 years

We hope you enjoyed reading the articles published last year. We already have enough leading articles for 2010. We had a successful year in 2009, with over 266 submissions. The number of submissions is increasing year by year[Figure 1]. These articles of course will be available online well before their print publication date. Phcog Mag. is known for publishing the issue on time. Our article has passed the litmus test and it is now been indexed in most of the databases such as CAB Abstracts, Caspur, Chemical Abstracts, CSA databases, DOAJ, EBSCO Publishing’s Electronic Databases, Excerpta Medica/EMBASE, Google Scholar, Hinari, Index Copernicus, Indian Science Abstracts, Journal Citation Reports, OpenJGate, PrimoCentral, ProQuest, PubMed, Pubmed Central, Science Citation Index Expanded, SCOLOAR, SCOPUS, SIIC databases, Summon by Serial Solutions, Ulrich’s International Periodical Directory, Web of Science, etc.

## PEER REVIEW PROCESS

On behalf of the Editor-in-Chief and editorial board members, I would like to thank all the reviewers who have helped us in refining the papers submitted to Phcog Mag. We acknowledge them fully for their contribution and we have published a list of all of our reviewers in page 81. Table 2 summarizes the acceptance/ratio percentage. Performance of our reviewers is included in Table 3. We are constantly adding new reviewers. Hopefully, we will have large number of active and dedicated reviewers by this year end. Our reviewers review the papers for us on time. Not all the time, our reviewers’ response toward my invitation is fruitful. Most of our reviewers remain busy with their busy research or academic schedules. We are extremely thankful to them for spending their precious time in refining the articles of Phcog Mag.

**Table 2:** Number of Articles submitted along with accepted/rejected ratio during the year 2010

Article type	Submitted	Accepted	Rejected	Under review
Editorial	8	1 (13)	7 (88)	0 (0)
Guest editorial	1	1 (100)	0 (0)	0 (0)
Letter to editor	3	2 (67)	1 (33)	0 (0)
Original article	240	68 (28)	136 (57)	36 (15)
Short communication	14	2 (14)	10 (71)	2 (14)
Total decisions	228	74 (32)	154 (68)	
Total articles	266	74 (28)	154 (58)	

Courtesy: Journalonweb, Figures in parentheses are in percentage

**Table 3:** Reviewers’ performance for the year 2010

Total referees	167
Number of reviewers used (/article)	122 (2)
Number of reviewers responded (/article)	101 (1)
Reviewers on time	135 (111%)
Returned late	67 (55%)
Never returned	46 (38%)
Required reminders	0

Courtesy: Journalonweb

## PROCESSING TIME OF MANUSCRIPTS

The manuscripts are now peer reviewed using the powerful manuscript handling system, Journalonweb (Courtesy: Medknow Publications). The processing time of the manuscript is improving day by day. As can be seen in Table 4, the number of days to suggest and add reviewers is within a week’s time. Table 4 also shows the Editor’s response toward the reviewers, author’s reply, and the publication time. The number of days utilized for a paper is now approximately around 106 days from the time of submission. The total period of publication time from the time of submission is now approximately 56 days. We will try to reduce the time in the coming years by speeding the pagination and editorial/peer review process.

**Table 4:** Submitted to first decision for the year 2010

Number of articles submitted	266
Days to suggest reviewers	7.63 (0, 51)
Days taken by reviewers	21.44 (1, 42)
Days taken by the Editor for decision	7.50 (0, 78)
Days until paper is under review	36.57
Revisions for the year 2010	
Days from first decision until revision arrives	14.82 (0, 79)
Days for the Editor to take decision	1.00 (0, 1)
Days to send revision decision by the Editors	1.00 (0, 1)
Days from revision receipt to revision decision	2
Accepted papers for the year 2010	
Days from first submission to acceptance	106.40 (0, 198)
Days from acceptance to publication	56.35 (9, 119)

Courtesy: Journalonweb

[Table 5] summarizes the number of authors registered in manuscript submission site. As can be seen, more and more papers are being submitted from abroad.

**Table 5:** Results for the year 2010

Total number of authors registered with the site	337
Number of authors who have submitted manuscripts	215
Number of authors who have submitted more than one manuscript	10
Number of manuscripts from abroad	143 (54%)
Number of original articles from abroad	134 (56%)

Courtesy: Journalonweb

## CHALLENGES AHEAD

We are constantly working on the quality of articles published in each issue. From January 2011, we have restricted to publish the content covering preliminary studies, etc.[1] Further, based on authors’ need, we have included many article types from this year.

There is a drastic rise in the article downloads from the website and we are expecting that more number of articles will be cited in this year. Phcog Mag. SNIP value for the year 2009 is 0.260 (Courtesy: SCOPUS). It is likely to have Scmiago Journal Ranking (SJR) as we are already indexed in SCOPUS and we are also expecting to have an Impact Factor at the end of this year and I would like to list here the most cited articles from 2008.[2–25] Finally, I would like to inform that the publishing cost is very high. As we are not still self-sufficient to cover the cost, we request all the authors to subscribe the journal and strengthen us financially.

I wish you a Happy New Year!

## References

[1] Mueen Ahmed KK (2010). Major changes in Pharmacognosy Magazine. Phcog Mag [serial online].

[2] Ebrahimzadeh M, Ehsanifar S, Eslami B *Sambucus ebulus elburensis* fruits: A good source for antioxidants [Internet]. http://www.phcog.com/text.asp?2009/5/19/213/58162.

[3] Nabavi S, Ebrahimzadeh M, Nabavi S, Fazelian M, Eslami B *In vitro* Antioxidant and Free Radical Scavenging Activity of *Diospyros lotus* and *Pyrus boissieriana* growing in Iran [Internet]. http://www.phcog.com/text.asp?2009/5/18/122/57969.

[4] Raju K, Balaraman R Antidiabetic mechanisms of Saponins of *Momordica cymbalaria* [Internet]. http://www.phcog.com/text.asp?2008/4/15/197/58031.

[5] Shirode D, Patel T, Roy S, Jyothi T, Rajendra S, Prabhu K Anti-ulcer properties of 70% ethanolic extract of leaves of *Albizzia lebbeck* [Internet]. http://www.phcog.com/text.asp?2008/4/15/228/58043.

[6] Kumar A, Mazumder A, Saravanan V Antihyperlipidemic activity of *Camellia sinensis* leaves in Triton WR-1339 induced albino rats [Internet]. http://www.phcog.com/text.asp?2008/4/13/60/58066.

[7] Roy B, Lalchhandama K, Dutta B Scanning electron microscopic observations on the *in vitro* anthelmintic effects of *Millettia pachycarpa* on *Raillietina echinobothrida* [Internet]. http://www.phcog.com/text.asp?2008/4/13/20/58054.

[8] Sureshkumar S, Mishra S In-vitro evaluation of hepatoprotective activity of *Pergularia daemia* Forsk [Internet]. http://www.phcog.com/text.asp?2008/4/16/298/58002.

[9] Mruthunjaya K, Hukkeri V *In vitro* Antioxidant and free radical scavenging potential of *Parkinsonia aculeata* Linn [Internet]. http://www.phcog.com/text.asp?2008/4/13/42/58060.

[10] Nanu R, Raghuveer I, Chitme H, Chandra R Antidiabetic Activity of *Nyctanthes arbortristis* [Internet]. http://www.phcog.com/text.asp?2008/4/16/335/58010.

[11] Jayakody JR, Ratnasooriya W Blood Glucose Level Lowering Activity of Sri Lankan Black Tea brew (*Camellia sinensis*) in rats [Internet]. http://www.phcog.com/text.asp?2008/4/16/341/58012.

[12] Kapoor S, Saraf S Efficacy Study of Sunscreens Containing Various Herbs for Protecting Skin from UVA and UVB Sunrays [Internet]. http://www.phcog.com/text.asp?2009/5/19/238/58167.

[13] Garg M, Dhar V, Kalia A Antidiabetic and antioxidant potential of *Phyllanthus fraternus* in alloxan induced diabetic animals [Internet]. http://www.phcog.com/text.asp?2008/4/14/138/58582.

[14] Suthar A, Mulani R A high performance thin layer chromatography method for quantitative estimation of Diosgenin in *Solanum nigrum* Linn. [Internet]. http://www.phcog.com/text.asp?2008/4/14/112/58578.

[15] Kostadinova E, Alipieva K, Stefova M, Antonova D, Evstatieva L, Stefkov G Influence of cultivation on the chemical composition and antimicrobial activity of *Sideritis* spp. [Internet]. http://www.phcog.com/text.asp?2008/4/14/102/58576.

[16] Dhasan P, Jegadeesan M, Kavimani S Cucurbitacins isolated from the fruits of *Momordica cymbalaria* Hook f. [Internet]. http://www.phcog.com/text.asp?2008/4/14/96/58575.

[17] Amudhan M, Begum V Alpha-glucosidase inhibitory and hypoglycemic activities of *Areca catechu* extract [Internet]. http://www.phcog.com/text.asp?2008/4/15/223/58042.

[18] Pattanayak S, Priyashree S Hepatoprotective activity of the leaf extracts from *Dendrophthoe falcata* (L.f) Ettingsh against carbon tetrachloride-induced toxicity in wistar albino rats [Internet]. http://www.phcog.com/text.asp?2008/4/15/218/58041.

[19] Shahverdi A, Ostad S, Khodaee S, Bitarafan L, Monsef-Esfahani H, Jamalifar H Antimicrobial and cytotoxicity potential of *Peganum harmala* smoke [Internet]. http://www.phcog.com/text.asp?2008/4/15/236/58046u.

[20] Singh R, Madan J, Rao H Antiulcer Activity of Black Pepper against Absolute Ethanol Induced Gastric Mucosal Damage in Mice [Internet]. http://www.phcog.com/text.asp?2008/4/15/232/58044.

[21] Shrinivas S, Ravindra K, Aradhana M, Kailash B Ethanolic leaf extract of *Holoptelea integrifolia*, Planch. decreases cisplatininduced pica in rats [Internet]. http://www.phcog.com/text.asp?2008/4/16/293/58001.

[22] Ionkova I, Fuss E Influence of different strains of *Agrobacterium rhizogenes* on induction of hairy roots and lignan production in *Linum tauricum* ssp. tauricum [Internet]. http://www.phcog.com/text.asp?2009/5/17/14/57980.

[23] Omwenga E, Okemo P, Mbugua P, Ogol CK Ethnobotanical Survey and Antimicrobial Evaluation of Medicinal Plants used by the Samburu Community (Kenya) for treatment of Diarrhorea [Internet]. http://www.phcog.com/text.asp?2009/5/18/165/57976.

[24] Rathod N, Raghuveer I, Chitme H, Ramesh C Prevention of high-fructose diet induced insulin resistance by *Nyctanthes arbortristis* and *Calotropis gigantea* in rats [Internet]. http://www.phcog.com/text.asp?2009/5/19/58/59784.

[25] Shokrzadeh M, Saeedi Saravi S, Mirzayi M Cytotoxic Effects of Ethyl Acetate Extract of *Sambucus ebulus* Compared with Etoposide on Normal and Cancer Cell Lines [Internet]. http://www.phcog.com/text.asp?2009/5/20/316/58152.

